# Definition and management of very high fracture risk in women with postmenopausal osteoporosis: a position statement from the Brazilian Society of Endocrinology and Metabolism (SBEM) and the Brazilian Association of Bone Assessment and Metabolism (ABRASSO)

**DOI:** 10.20945/2359-3997000000522

**Published:** 2022-10-03

**Authors:** Barbara C. Silva, Miguel Madeira, Catarina Brasil d’Alva, Sergio Setsuo Maeda, Narriane Chaves Pereira de Holanda, Monique Nakayama Ohe, Vera Szejnfeld, Cristiano A. F. Zerbini, Francisco José Albuquerque de Paula, Francisco Bandeira

**Affiliations:** 1 Santa Casa de Belo Horizonte Unidade de Endocrinologia Belo Horizonte MG Brasil Unidade de Endocrinologia, Santa Casa de Belo Horizonte, Belo Horizonte, MG, Brasil; 2 Hospital Felício Rocho Unidade de Endocrinologia Belo Horizonte MG Brasil Unidade de Endocrinologia, Hospital Felício Rocho, Belo Horizonte, MG, Brasil; 3 Centro Universitário de Belo Horizonte Departamento de Medicina Belo Horizonte MG Brasil Departamento de Medicina, Centro Universitário de Belo Horizonte (UNI-BH), Belo Horizonte, MG, Brasil; 4 Universidade Federal do Rio de Janeiro Divisão de Endocrinologia e Metabolismo Rio de Janeiro RJ Brasil Divisão de Endocrinologia e Metabolismo, Universidade Federal do Rio de Janeiro, Rio de Janeiro, RJ, Brasil; 5 Universidade Federal do Ceará Departamento de Medicina Clínica Fortaleza CE Brasil Departamento de Medicina Clínica, Universidade Federal do Ceará (UFC), Fortaleza, CE, Brasil; 6 Universidade Federal de São Paulo Escola Paulista de Medicina Unidade de Endocrinologia São Paulo SP Brasil Unidade de Endocrinologia, Escola Paulista de Medicina, Universidade Federal de São Paulo, São Paulo, SP, Brasil; 7 Universidade Federal da Paraíba Divisão de Endocrinologia e Metabolismo João Pessoa PB Brasil Divisão de Endocrinologia e Metabolismo, Universidade Federal da Paraíba, João Pessoa, PB, Brasil; 8 Universidade Federal de São Paulo Escola Paulista de Medicina Divisão de Reumatologia São Paulo SP Brasil Divisão de Reumatologia, Escola Paulista de Medicina, Universidade Federal de São Paulo, São Paulo, SP, Brasil; 9 Centro Paulista de Investigação Clínica São Paulo SP Brasil Centro Paulista de Investigação Clínica, São Paulo, SP, Brasil; 10 Universidade de São Paulo Faculdade de Medicina de Ribeirão Preto Departamento de Clínica Médica Ribeirão Preto SP Brasil Departamento de Clínica Médica, Faculdade de Medicina de Ribeirão Preto, Universidade de São Paulo, Ribeirão Preto, SP, Brasil; 11 Universidade de Pernambuco Faculdade de Medicina Divisão de Endocrinologia e Metabolismo Recife PE Brasil Divisão de Endocrinologia e Metabolismo, Faculdade de Medicina, Universidade de Pernambuco, Recife, PE, Brasil

**Keywords:** Osteoporosis, very high risk of fracture, anabolic, teriparatide, romosozumab

## Abstract

Several drugs are available for the treatment of osteoporosis in postmenopausal women. Over the last decades, most patients requiring pharmacological intervention were offered antiresorptive drugs as first-line therapy, while anabolic agents were considered a last resource for those with therapeutic failure. However, recent randomized trials in patients with severe osteoporosis have shown that anabolic agents reduce fractures to a greater extent than antiresorptive medications. Additionally, evidence indicates that increases in bone mineral density (BMD) are maximized when patients are treated with anabolic agents first, followed by antiresorptive therapy. This evidence is key, considering that greater increases in BMD during osteoporosis treatment are associated with a more pronounced reduction in fracture risk. Thus, international guidelines have recently proposed an individualized approach to osteoporosis treatment based on fracture risk stratification, in which the stratification risk has been refined to include a category of patients at very high risk of fracture who should be managed with anabolic agents as first-line therapy. In this document, the Brazilian Society of Endocrinology and Metabolism and the Brazilian Association of Bone Assessment and Metabolism propose the definition of very high risk of osteoporotic fracture in postmenopausal women, for whom anabolic agents should be considered as first-line therapy. This document also reviews the factors associated with increased fracture risk, trials comparing anabolic versus antiresorptive agents, efficacy of anabolic agents in patients who are treatment naïve versus those previously treated with antiresorptive agents, and safety of anabolic agents.

## INTRODUCTION

In many chronic diseases, therapeutic inertia causes a considerable gap between recommended guidelines and treatment implementation in clinical practice (1). The treatment gap in osteoporosis leads to many preventable fractures that occur without appropriate treatment (2). After an episode of hip fracture, the rate of treatment initiation to prevent future fractures is low (3), despite robust evidence of a better outcome with pharmacological intervention (4).

Screening for osteoporosis is crucial for identifying high-risk patients who would benefit most from pharmacological treatment (5). In the SCOOP study, 12,483 postmenopausal women aged 70-85 years were randomized to a screening program using the Fracture Risk Assessment Tool (FRAX) or usual management (6). The women were identified from primary care centers and, in those at high risk of fractures, treatment was recommended. At the end of the first year, 15.3% of the participants in the screening group were on pharmacological treatment, compared with 4.5% of those in the control group. Over 5 years of follow-up, there was a 28% reduction in the rate of hip fracture in the screening group (hazard ratio [HR]: 0.72, 95% confidence interval [CI] 0.59-0.89; p = 0.002). This reduction was greater in women with a baseline FRAX probability of hip fracture in the 90th percentile (HR: 0.67, 95% CI 0.53-0.84; p = 0.002) (7). Among patients who were classified as being at high risk of fracture in the screening arm and who self-reported use of antiosteoporotic medication at 6 months, 38.2% remained on treatment at 60 months compared with 21.6% of controls (8).

In a recent meta-analysis of randomized studies evaluating the role of population screening for postmenopausal osteoporosis in preventing fracture, data from the SCOOP trial were combined with data from two other studies, *i.e.*, the ROSE and SOS studies, including 18,605 and 11,032 patients, respectively, aged 65-90 years (9). The FRAX probability of fracture was also used to identify high-risk patients (9). The proportion of patients who started pharmacological treatment and the mean follow-up period were, respectively, 11% and 5 years in ROSE, 15% and 4.8 years in SCOOP, and 18% and 3.7 years in SOS. There were significant reductions in the rates of major osteoporotic (HR: 0.91, 95% CI 0.84-0.98) and hip (HR: 0.80, 95% CI 0.71-0.91) fractures in screened patients compared with controls receiving usual care (9). These data reinforce the need for proper implementation of screening programs to reduce the fracture burden.

Several organizations have recommended risk stratification at screening as an important tool in osteoporosis management (10-14). These recently published guidelines have refined the fracture risk stratification to include a category of patients at a very high risk of fracture (Table 1). Over the last decades, most patients requiring pharmacological intervention were offered antiresorptive drugs as first-line therapy, whereas anabolic agents were considered as last resource for those with therapeutic failure. Although bisphosphonates continue to be the first-line agents for long-term pharmacological therapy in high-risk patients (15), recent randomized trials comparing bone-forming agents with bisphosphonates have shown that anabolic agents reduce fractures to a greater extent than antiresorptive agents in patients with severe osteoporosis (16,17). Additionally, it became apparent that the reduction in fracture risk may be more pronounced when patients are treated with anabolic agents first, followed by an antiresorptive drug (18,19). In a meta-regression analysis of 38 randomized controlled trials of antiosteoporotic medications, Bouxsein and cols. (19) found that a 6% increase in total hip (TH) bone mineral density (BMD) was associated with a 66% reduction in vertebral fractures and 40% reduction in hip fractures. In this regard, for patients in the “very high risk” category (those who have a greater immediate risk of future fracture), starting with an anabolic followed by an antiresorptive agent would induce greater increases in femoral neck (FN) and TH BMD, which have been associated with a more pronounced reduction in fracture risk at any site, therefore avoiding a larger number of fracture than the single-antiresorptive strategy (13).

**Table 1:** Definition criteria of very high fracture risk according to different organizations

Criteria/Organization	AACE Camacho and cols.,2020 (10)	ES Eastell and cols., 2019, and Shoback and cols., 2020 (12,14)	ESCEO/IOF Kanis and cols., 2020 (13)	NICE/NOGG Compston and cols., 2017 (11)
Recent fracture	<12 months	-	<12 months	<12 months
Fracture while on approved therapy	+	-	-	-
Drugs causing skeletal harm	Fracture on long-term glucocorticoids	-	Long-term high-dose glucocorticoids* plus T-score < -2.0	-
Multiple fractures	+	Vertebral	-	Vertebral
Low BMD	T-score < -3	T-score ≤ - 2.5 and prevalent fractures	-	-
High risk of falls or history of injurious falls	+	-	-	-
High FRAX probability	MOF > 30% Hip fracture > 4.5%	-	Age-specific threshold for MOF**	-

AACE: American Association of Clinical Endocrinology; ES: Endocrine Society; ESCEO: European Society for Clinical and Economic Aspects of Osteoporosis, Osteoarthritis and Musculoskeletal Diseases; IOF: International Osteoporosis Foundation; NICE: National Institute of Clinical Excellence; NOGG: National Osteoporosis Guidelines Group; BMD: bone mineral density; MOF: major osteoporotic fractures.

* >5 mg/day of prednisone or equivalent.

** 50-54 years: >9.4%; 55-59 years: >13.2%; 60-64 years: 16.8%; 65-69 years: 22.8%; 70-74 years: 26.4%; 75-79 years: 31.2%; 80-84 years: 37.2%; 85-89 years: >39.6%.

The aim of this document is to provide clinicians with a position from experts at the Brazilian Society of Endocrinology and Metabolism (SBEM) and the Brazilian Association of Bone Assessment and Metabolism (ABRASSO) on the evidence-based definition of very high fracture risk in postmenopausal women. It also aims to describe for whom anabolic agents should be considered as first-line therapy. The document also reviews the factors associated with increased fracture risk, the comparative trials of anabolic and antiresorptive agents, the efficacy of anabolic agents in patients who are treatment-naïve versus those previously treated with antiresorptive medications, and the safety of anabolic agents.

## MATERIALS AND METHODS

Members of SBEM and ABRASSO performed a narrative review based on a rigorous literature search using PubMed. The literature search was limited to the English language and human subjects, and included original articles, systematic and narrative reviews, and guidelines by medical societies published between 2000 and 2021, as well as pertinent articles from authors’ libraries. Relevant articles were reviewed in detail. Panel members reached consensus after several virtual meetings to discuss the available data and finalize the non-graded recommendations presented in this review.

### Clinical and densitometric findings associated with increased fracture risk

#### Recent and multiple fragility fractures

A prior fragility fracture is a very important, if not the main, risk factor for subsequent fractures. The time elapsed since the prior fracture, as well as characteristics specific to this fracture, are now recognized as important factors influencing subsequent risk. In a large cohort of 18,872 individuals, the risk of a second major osteoporotic fracture (MOF) was 2.7-fold higher than the risk of fracture in the background population within the first year following a MOF, and declined thereafter, yet not to the rates of the general population. In individuals with an incident fracture, a refracture occurred in 20% within 1 year and 34% within 2 years (20). The observation that the risk of a new fracture declines over time following an index fracture is in agreement with several studies (21-23) demonstrating up to 5-fold higher fracture risk within 1 year post-fracture (24).

Virtually all types of fragility fractures, including fractures in nonvertebral sites such as wrist and humerus, are followed by imminent fracture risk (25-27), but the magnitude of the risk of refracture varies by the location of the previous fracture (Table 2). In three recent large cohorts of patients with an incident fracture, the incidence of subsequent fractures within 1-2 years was highest in those with an index vertebral fracture (28-30). Patients with an index wrist fracture had lower absolute risk relative to those with index fracture occurring at most of the other skeletal sites (29). The risk was lowest following prior tibia/fibula and ankle fracture (29,30). Importantly, the proportion of patients with a fragility fracture who fractured again increased with age in all these studies. Further, the risk of new vertebral fractures increases progressively with the number of prior vertebral fractures (31-33). In a large sample of participants of the Fracture Interventional Trial with prevalent vertebral fractures, the incidence of new vertebral fracture increased from 3.8% in women without prior vertebral fracture to 8.9%, 19.4%, 30.8%, or 54.2% in those with 1, 2, 3-4, or 5 or more prevalent vertebral fractures, respectively (33). Association between prior nonvertebral fractures and the risk of subsequent fractures was also investigated; in a cohort of 51,762 women, the subsequent 2-year fracture incidence was 10%, 16%, and 25% for women with one, two, or three or more prior fractures at any of 10 different skeletal sites. The increased risk was most dramatic for spine fractures; a history of ≥ 3 prior fractures carried a 9-fold risk of a subsequent vertebral fracture (34). Therefore, a recent fracture (within the past 2 years) is a predictor of imminent fracture risk and multiple fragility fractures; even when the fractures occurred remotely, they are associated with a very high risk of near-term fracture.

**Table 2:** Incidence of subsequent fractures by type of index fragility fracture in different cohorts

Study	Number of patients with an index fracture/age	Incidence (%) of subsequent fractures	Incidence of any subsequent fracture by type of index fracture
Toth (Sweden), 2020 (28)	35,146 55-90 years	11.3% (24 months)	Vertebral (17.6%) > hip (13.7%) > humerus (11.4%) > wrist, forearm, and others (data not shown)
Adachi (Canada), 2021(29)	115,776 ≥65 years	17.8% (median time 555 days)	Vertebral (23%) > pelvis (21.3%) > radius/ulna (20.2%) > multisite (20.1%) > humerus (18.9%) > clavicle/ribs/sternum (18.7%) > wrist (17.7%) > femur (16.0%) > hip (15.9%) > tibia/fibula/knee (13.4%)
Balasubramanian (US), 2019 (30)	210,621 ≥65 years	17.9% (2 years)	Spine (25.5%) > pelvis (20.2%) > clavicle (18.3%) > humerus (15.7%) > hip (15.0%) > femur (13.9%) > radius/ulna (13.9%) > tibia/fibula (12.1%) > ankle (9.5%)

#### Bone mineral density T-score lower than -3.0 standard deviations

There is a strong inverse relationship between BMD and risk of fractures. For every 1 standard deviation (SD) decrease in age-adjusted BMD, the relative risk (RR) of fracture increases 1.6- to 2.6-fold (35). A low BMD is also a risk factor of imminent fracture (*i.e.*, fracture within 1-2 years). A retrospective study of 3,228 women aged > 65 years from the Canadian Multicentre Osteoporosis Study identified four independent predictors of 2-year low-trauma nonvertebral fractures, including a T-score ≤ -3.5 at the TH (HR: 3.7; p < 0.001), two or more falls in the previous year (HR: 1.9; p < 0.001), at least one low-trauma fracture in the previous year (HR: 1.7; p = 0.055), and a low score in the physical component of the SF-36 quality of life questionnaire (HR: 1.6; p < 0.001) (36). Similar predictors of 1-year nonvertebral fractures were identified in a cohort of 1,470 women aged > 65 years from the Framingham Study original cohort, namely a hip T-score ≤ -2.5 (HR: 2.8; p < 0.001), a poor self-rated health (HR: 4.0; p = 0.04), and the use of nitrates in the previous year (HR: 2.6; p = 0.01) (37).

Thus, low BMD is a major predictor not only of long-term fracture risk (38) but also of imminent fracture. Since this concept of imminent fracture is central to the categorization of very high risk of fracture, one might consider a low T-score as an isolated criterion to define a very high risk of fracture. In fact, the recent guidelines of the American Association of Clinical Endocrinology (AACE) include a T-score ≤ -3.0 SD as characterizing patients at very high risk of fracture (10). Other authors suggest a T-score ≤ -3.5 SD as a criterion to define a very high fracture risk (39). However, especially for the imminent fracture risk, factors other than BMD are extremely important, such as advanced age, previous fracture, and high falling risk, among others (29,36,37,40). Thus, the present position statement considers that an isolated T-score ≤ -3.0 SD (without other predictive factors such as a prior fracture, advanced age, high risk of fall, or glucocorticoid use) may not be indicative of a very high fracture risk.

#### High risk of falls

Falls are the leading cause of injury-related morbidity and mortality among older adults (41), and over 50% of deaths due to falls are a result of complications following a hip fracture (42). A systematic review by Ganz and cols. concluded that older adults with a history of falls are likely to fall again (likelihood ratio [LR] 2.3-2.8), and that the most consistent predictor of future falls was a clinically detected gait or balance abnormality (LR 1.2-2.4) (43). Data on risk factors obtained from meta-analyses of observational studies have revealed the following odds ratios (ORs) and 95% CIs for any falls (44): gait problems, 2.06 (1.82-2.33) (45); balance impairment, 1.98 (1.6-2.46) (46); physical disability, 1.56 (1.22-1.99) (45); orthostatic hypotension, 1.50 (1.15-1.97) (47); depressive symptoms, 1.49 (1.24-1.79) (48); visual impairment, 1.35 (1.18-1.54) (45); and cognitive impairment, 1.32 (1.18-1.49) (49). The following medications are likewise related to risk factors for falls: antipsychotics, 2.30 (1.24-6.26) (50); antidepressants, 1.48 (1.24-1.77); benzodiazepines, 1.40 (1.18-1.66); and polypharmacy, 1.75 (1.27-2.41). Some of these factors increase not only the risk of falls but also the risk of fracture (40,51). In addition, the history of fall itself is a risk for imminent fracture (36,40,51). As previously mentioned, two or more falls in the previous year almost doubled the risk of osteoporotic nonvertebral fracture (HR: 1.9; p < 0.001) in the following 2 years in women aged > 65 years from the Canadian Multicentre Osteoporosis Study (36). A history of at least one fall in the previous 1 or 2 years also increased the risk of imminent fracture in two additional cohorts of older people (mostly women), with risk ratios ranging from 2.2 to 6.7 (40,51).

Therefore, it is important to consider the risk factors for falls in the previous year in the evaluation of patients at risk for fracture, since these factors are associated with imminent risk of fracture and may be useful to classify elderly women in the category of very high fracture risk. The present position statement considers that older women at high risk of falls are those who have had two or more falls in the previous year, or those with decreased physical and cognitive performance, with a high frailty status.

#### Risk stratification estimated by FRAX

FRAX is the most used fracture risk calculator incorporated into guidelines for the management of osteoporosis. In Brazil, as in many countries of Europe and Latin America, the approach used to define pharmacological treatment intervention with FRAX is based on an age-dependent intervention threshold adopted by the National Osteoporosis Guideline Group (NOGG) (52-55). Recently, the International Osteoporosis Foundation and European Society for Clinical and Economic Aspects of Osteoporosis and Osteoarthritis (IOF-ESCEO) have published guidance recommending the use of FRAX to define a very high fracture risk (13). Using fully age-dependent intervention thresholds, a fracture probability calculated with or without BMD that exceeds the intervention threshold by 20% would categorize individuals at very high fracture risk. Thus, individuals eligible for treatment are categorized as being at high or very high risk of fracture if their fracture probability falls, respectively, below or above 1.2 times their age-specific intervention threshold (13).

This recommendation, however, may classify too many individuals into the category of very high fracture risk when the hybrid intervention threshold is used (56). Over the last few years, NOGG has adopted a “hybrid approach” defined as an age-dependent threshold up to the age of 70 years, with a fixed threshold thereafter (11,57), reducing the dependence on the existence of a prior fracture after this age. Recently, a NOGG working group has compared the impact of setting an upper intervention threshold (UIT) equivalent to 1.2, 1.6, and 2.0 times the preexisting age-dependent intervention threshold in a simulated UK population of women aged 55-90 years, to distinguish the “very high” from the “high” risk categories using the FRAX hybrid model (56). Among women eligible for treatment, the proportion of those at very high fracture risk was 55.7% (too high), 25.1%, and 12.1% (very low) using the UITs of 1.2, 1.6, and 2.0, respectively. The authors considered that a UIT of 1.6 would be more appropriate in the hybrid model. Indeed, using the UIT of 1.6, most participants in phase 3 trials of romosozumab and teriparatide (TPTD) would fall in the very-high-risk category (56).

The use of FRAX to identify individuals who are at very high risk of fracture has been questioned by some authors (58). The current version of FRAX does not consider the recency, site, or number of prior fractures − factors that have been shown to be important determinants of risk for imminent fractures (58). In addition, FRAX does not consider the risk of falls or the age when the fractures occurred. On the other hand, a recent *post hoc* analysis of the FRAME study has shown that, compared with placebo, the efficacy of romosozumab in reducing clinical, osteoporotic, and MOFs was greater in women with a higher FRAX probability of fracture at baseline (59). These data suggest that FRAX could be useful in identifying better responders to this anabolic agent.

The present position statement considers that, while the categorization of patients at very high risk of fracture should not be based solely on FRAX, this tool can be used as one of the criteria to identify these patients. The UIT of 1.2 to define patients at very high risk of fracture, as recommended by the IOF-ESCEO, appears to be appropriate for adoption in Brazil, since the FRAX Brazil uses the fully age-dependent intervention threshold rather than the hybrid model.

#### Fracture while on prolonged use of glucocorticoid

Glucocorticoid-induced osteoporosis (GIO) is the most common form of secondary osteoporosis. Rapid bone loss and increased fracture risk may occur within the first 6 months of continuous use of glucocorticoid (GC), while fracture risk remains high during treatment, decreasing only after GC withdrawal (60). The risk of fracture in GIO is higher than in postmenopausal or senile osteoporosis at the same values of BMD (61). Vertebral fractures are more common in GIO, but nonvertebral and hip fractures may also occur (61). The risk of fracture is significantly associated with GC dose. Daily doses of prednisone < 2.5 mg, 2.5-7.5 mg, and > 7.5 mg lead to GIO RRs of 1.55, 2.59, and 5.18, respectively. Bone loss in GIO may lead to a very high risk of fracture (62).

#### Fracture while on antiosteoporotic treatment

The efficacy of the treatment response can be evaluated only in patients who have adhered to the treatment regimen (80% of doses taken) for at least 1 year, with calcium and vitamin D adequacy. Poor persistence is associated with loss of fracture risk reduction. Persistence is essential for the successful treatment of osteoporosis and can be improved by therapeutic education (63). In a systematic review, factors associated with poorer medication adherence included polypharmacy, older age, higher dosing frequency, medication side effects, and misconceptions about osteoporosis. The history of falls was associated with higher medication adherence (64). The IOF working group has defined that treatment failure in osteoporosis should be considered in the case of two or more incident fragility fractures (fractures of the hand, skull, digits, feet, and ankle are not considered as fragility fractures), significant bone loss (5% or more in at least two serial BMD measurements at the lumbar spine (LS) or 4% at the proximal femur), or nonresponse in bone turnover markers (decline of less than 25% from baseline levels for antiresorptive treatments, and less than 25% increase for TPTD after 6 months) in a patient who has been treated for more than 12 months and with no secondary causes of bone loss or fracture (65). The working group recommends that a weaker antiresorptive can be reasonably replaced by a more potent drug of the same class, an oral drug can be replaced by an injected drug, and a strong antiresorptive can be replaced by an anabolic agent (65).

More recently, the Endocrine Society suggested a greater change in bone turnover markers (~56% for serum C-telopeptide [CTX] and ~38% for procollagen type 1 N-terminal propeptide [P1NP]) to be considered an optimal response to treatment (12) and recommended that CTX or P1NP can be used to identify poor response or nonadherence to antiresorptive or anabolic therapy, respectively. While the authors agreed that having two or more fractures while on therapy is considered treatment failure, the occurrence of a single fracture in a compliant patient should raise the consideration for changing therapy (12). The AACE guideline for osteoporosis treatment suggests that the occurrence of fractures while on approved osteoporosis therapy is a criterion to define very high risk of fracture and recommends therapy with potent injected antiresorptive agents or anabolic drugs in these cases (10).

### Head-to-head comparative trials of anabolic versus antiresorptive agents

Two bone-forming agents approved for treatment of postmenopausal osteoporosis are currently available in Brazil: TPTD (1-34 aminopeptide of PTH or PTH[1-34]), the foreshortened fragment of PTH(1-84), and romosozumab, a monoclonal anti-sclerostin antibody. TPTD is an osteoanabolic agent that increases bone formation and resorption and is available in Brazil as a 20 µg subcutaneous (SC) daily dose, given for a maximum of 24 months. In contrast, romosozumab stimulates bone formation and inhibits bone resorption and is given as a 210 mg SC monthly dose for 12 months (66).

Anabolic therapy should be considered as initial treatment for very-high-risk patients. In these patients, rapid fracture risk reduction is required. Pivotal trial data and a few head-to-head studies indicate that anabolic agents produce a more rapid and greater reduction in nonvertebral fracture risk as well as a greater reduction in vertebral fracture risk compared with antiresorptive therapies over 1 to 2 years of treatment (described below) (16,17,67,68). However, these comparator studies utilized oral bisphosphonates (alendronate and risedronate) and not the most potent parenteral antiresorptive agents, denosumab or zoledronic acid.

#### Anabolic versus antiresorptive agents in postmenopausal women with low bone mineral density and previous fractures

A few anabolic/antiresorptive comparator clinical trials have evaluated fractures as primary outcomes, and they all included women with severe osteoporosis and prevalent vertebral or hip fracture (Table 3) (16,17,68). In the VERO study, the antifracture efficacy of TPTD was compared with that of risedronate in postmenopausal women with severe osteoporosis (16). TPTD therapy led to a lower risk of new vertebral and clinical fractures compared with risedronate. TPTD reduced the incidence of vertebral fracture by 50% within 1 year (p = 0.01) and resulted in a trend toward reduced nonvertebral fracture incidence (p = 0.099) over 2 years (16).

**Table 3:** Anabolic/antiresorptive comparator clinical trials that have considered fractures as primary outcomes

Study	Study design and treatment	Study population	Results
VERO Study Kendler and cols., 2018 (16)	2-year, randomized, double-blind, double-dummy, active-controlled, multi-country trial, comparing TPTD (SC, 20 µg daily) versus RIS (PO, 35 mg weekly).	1,360 PostM women (680 in each treatment group), mean age 72.1 ± 8.7 years; mean BMD T-score -2.3 at the LS, -2.3 at the FN, and -2.0 at the TH. 100% of participants had at least one VFx, of whom 65% had two or more VFx, and 36% had a VFx within 1 year before entering the study. ~43% had at least one nonvertebral Fx.	Primary endpoint: 6.6% absolute risk reduction of new radiographic VFx in the TPTD group (RR 0.44, 95% CI 0.29-0.68; p < 0.0001).
			Secondary endpoints: lower risk of fractures in the TPTD group, as follows:
			- New and worsened VFx: RR 0.46 (95% CI 0.31-0.68), p < 0.0001.
			- Pooled clinical fracture: RR 0.48 (95% CI 0.32-0.74), p = 0.0009.
			- Nonvertebral fragility fracture: RR 0.66 (95% CI 0.39-1.10), p = 0.10.
			- Major nonvertebral fragility fracture: RR 0.58 (95% CI 0.32-1.05), p = 0.06.
ARCH Study Saag and cols., 2017 (17)	2-yr randomized, active-controlled, multi-country trial. Patients were treated, with ROMO, 210 mg, SC, monthly, or ALN, 70 mg, PO, weekly, in a blinded fashion for 1 year, followed by open-label ALN in both groups for additional 2 years.	4,093 PostM women (2,046 and 2,047 in each treatment group), mean age 74.3 years; mean BMD T-score -3.0 at the LS, -2.9 at the FN, and -2.8 at the TH. 99% of the participants had at least one fracture, including 96% who had ≥ 1 VFx, ~38% who had at least one nonvertebral Fx, and ~9% who had sustained a hip fracture.	Primary endpoint (over 2 years): 5.7% absolute risk reduction of new VFx in the ROMO-ALN group compared with the ALN group (RR 0.52, 95% CI 0.40-0.66; p < 0.001), and 3.3% absolute RR of clinical fractures (RR 0.73, 95% CI 0.61-0.88; p < 0.001).
			Secondary endpoints (over 2 years): lower risk of fractures in the ROMO-ALN group as follows:
			- Nonvertebral fractures: RR 0.81 (95% CI 0.66-0.99); p = 0.04.
			- Hip fractures: RR 0.62 (95% CI 0.42-0.92); p = 0.02.
Hagino and cols., 2021 (68)	120-week prospective, randomized, open-label Japanese study, comparing 72 weeks of TPTD followed by 48 weeks of ALN versus ALN for 120 weeks. TPTD: SC, 56.5 µg/week; ALN: PO 5 mg/day or 35 mg/week; or IV 900 µg every 4 weeks.	985 women (489 in the TPTD and 496 in the ALN group); mean age of 81.5 years; mean BMD T-score of -2.3 at the LS. 68% of the participants had at least one VFx, ~13.5% had sustained a hip fracture.	Primary endpoint: morphometric VFx incidence was reduced in the TPTD group (RR 0.78, 95% CI 0.61-0.99; p = 0.04).
			Secondary endpoints: No difference in the incidence of clinical VFx, any fracture, progression of VFx.

RR: risk ratio; PostM: postmenopausal; BMD: bone mineral density; FN: femoral neck; TH: total hip; LS: lumbar spine; VFx: vertebral fracture; Fx: fracture; ALN: alendronate; RIS: risedronate; TPTD: teriparatide; ROMO: romosozumab; yr: year; SC: subcutaneous; PO: oral administration; IV: intravenous.

In the ARCH study, fracture risk reductions with romosozumab versus alendronate were already apparent at 12 months, when romosozumab reduced the incidence of nonvertebral and vertebral fracture by 26% (p = 0.06) and 37% (p = 0.003), respectively, compared with alendronate. At 24 months, the risk of new vertebral fracture was reduced by 48% (p < 0.001) in patients treated with romosozumab followed by alendronate, when compared with alendronate alone. Moreover, nonvertebral fractures were reduced by 19% (p < 0.04) and hip fractures were reduced by 38% (p < 0.02) in patients who received romosozumab followed by alendronate compared with alendronate alone (17).

A *post hoc* analysis of the ACTIVE and its extension trials suggested a similar superiority of abaloparatide when compared with alendronate (69). The rates of vertebral fracture with 18 months of abaloparatide in the ACTIVE study were 71% lower than those observed with 24 months of alendronate during the ACTIVE extension trial (70,71). A trend toward lower nonvertebral fracture incidence was also observed with abaloparatide versus alendronate (45% risk reduction; p = 0.11).

Hadji and cols. studied 710 patients with active back pain due to vertebral fractures who were randomized to receive TPTD versus risedronate for 1 year. The incidence of recurrent vertebral fractures was 9% with risedronate and 4% with TPTD (p < 0.01), but there was no difference in nonvertebral fracture incidence (72). However, this study was designed to evaluate the effect of TPTD versus risedronate in reducing back pain, and the incidence of new vertebral fractures was a prespecified exploratory outcome.

#### Anabolic versus antiresorptive agents in individuals on chronic use of glucocorticoid

The mechanisms through which GCs decrease bone strength are complex, but the most consistent skeletal effects of GC are to inhibit osteoblastic function and promote osteoblast apoptosis. GIO is a typical bone formation disease (73). From a biological point of view, particularly in those patients receiving high dosages of GCs over the long term, the use of agents acting on osteoblasts and exerting an anabolic effect on bone seems appropriate. Bone quality and quantity may be restored to a greater extent with these compounds compared with inhibitors of bone resorption (74). To date, the only available osteoanabolic agent tested in a randomized clinical trial on GIO is TPTD. A head-to-head comparison of TPTD versus alendronate has been conducted in 428 men and women (80% women) with a mean age of 56 years, taking a median prednisone equivalent dose of 7.5 mg/day for 1.2-1.5 years. The mean BMD T-score was -2.6 at the LS and -2.0 at the TH. Overall, 27% of the participants had prevalent morphometric vertebral fractures, and 43% had nonvertebral fractures at baseline. Of note, only 9.3% of the study patients had been previously treated with bisphosphonates. The primary outcome was the change in BMD at the LS. The results showed that subjects with GIO treated with TPTD for 36 months had greater increases in BMD and fewer new vertebral fractures than those treated with alendronate (0.6% vs. 6.1%, respectively, p = 0.004), although the overall number of fractures was small (75).

Geusens and cols. performed a head-to-head comparison of TPTD versus risedronate in subgroups of the VERO trial, including GC-treated patients at a prednisone-equivalent dose of 5 mg/day or above (representing 9.3% of the cohort). They found consistent reduction in vertebral fracture risk in the TPTD group compared with the risedronate group, even in this subgroup of GIO patients (76).

The use in GIO has not yet been established for other osteoanabolic agents, such as abaloparatide and romosozumab, despite their use being approved for the treatment of postmenopausal osteoporosis. However, due to its combined anabolic and antiresorptive effect on bone, romosozumab may eventually become a promising treatment option in GIO (77).

### Efficacy of bone-forming agents in treatment-naïve patients and in individuals previously treated with antiresorptive drugs

The prescription of antiresorptive drugs is a *sine qua non* after a cycle of anabolic drugs. On the other hand, there is a heterogeneous response to anabolic drugs after antiresorptive therapy. When TPTD treatment follows the use of less potent antiresorptive agents, such as hormone therapy or raloxifene, the TPTD-induced increase in BMD appears to be preserved (18,78). Differently, TPTD prescribed after alendronate leads to a transient bone loss in proximal femur and only a slight beneficial effect in BMD at this bone site following 2 years of therapy (18,79). However, in recent randomized clinical trials and meta-analyses, TPTD and abaloparatide have demonstrated increases in BMD at both trabecular and cortical sites, resulting in fracture risk reduction regardless of prior bisphosphonate treatment (16,76,80). Romosozumab following bisphosphonate therapy has demonstrated BMD gains at the LS and hip sites, although more modest than in treatment-naïve patients (81,82). A randomized trial comparing TPTD with romosozumab therapy for 12 months in postmenopausal women previously treated with bisphosphonates (mostly alendronate) for a mean period of 6 years has shown significant greater increments in BMD at the LS, TH, and FN with romosozumab than TPTD (83). This study, however, did not assess the difference in fracture incidence between the groups (83).

The transition from denosumab to anabolic therapy has the potential to increase bone turnover and decrease bone mass. In the DATA-Switch study, the use of TPTD after denosumab led to marked bone loss in proximal femur, which was attenuated during the second year of TPTD treatment. However, there was continuing bone loss at the 1/3 radius (84). There are no data on fracture risk in patients treated with denosumab followed by TPTD, but a recently published position statement on the management of discontinuation of denosumab therapy discourages monotherapy with TPTD after denosumab (85).

Kendler and cols. have shown in a small study, designed to evaluate a three-cycle therapy (romosozumab-denosumab-romosozumab regimen), that after 12 months of denosumab treatment, another romosozumab cycle promoted a small increase in BMD at the LS and maintenance of BMD at the TH (86). Two observational studies have shown that subjects with osteoporosis treated for 1 year with romosozumab after treatment with denosumab for a mean duration of 24 to 39 months presented significantly lower gains in LS and hip BMD than treatment-naïve subjects (81,82). Although romosozumab decreased bone resorption in treatment-naïve individuals, the opposite finding of increased level of bone resorption marker was observed in patients previously treated with denosumab, which may have blunted BMD improvements (81,82). No data are available on fracture risk in patients treated with romosozumab following denosumab therapy for longer periods (>2.5 years), and the safety of transitioning from long-term use of denosumab to romosozumab is unknown.

### Ideal treatment sequences for patients at very high fracture risk

The aforementioned data suggest that the common practice of switching to bone-forming agents only after patients have an inadequate response to antiresorptive agents may not be the best approach to manage patients with severe osteoporosis. Indeed, there is evidence favoring the use of anabolic agents followed by potent antiresorptive therapy in patients at very high risk of fracture. Pivotal studies of anabolic and more potent parenteral antiresorptive agents suggest that the use of bone-forming compared with antiresorptive agents leads to a more rapid effect against all fractures, which is crucial in the management of patients with imminent risk of fractures (87-90). Moreover, recent data suggest that the use of anabolic followed by antiresorptive agents allows for greater increases in BMD (91,92). This is important considering new evidence showing that greater on-treatment increases in BMD, particularly at the femur, result in greater reduction in fracture risk (19,91,93,94).

### Definition and management of very high fracture risk in postmenopausal women

#### Treatment-naïve women

Postmenopausal women with osteoporosis presenting any of the following criteria should be classified in the very-high-risk category and be considered for anabolic therapy as initial treatment:

1. BMD T-score ≤ -2.5 at the LS, FN, or TH associated with at least one osteoporotic vertebral fracture OR a fragility hip fracture, particularly when the fracture occurred within the last 24 months.
2. Multiple osteoporotic vertebral fractures OR two or more nonvertebral osteoporotic fractures, regardless of the skeletal site and the BMD T-score, even when the fractures occurred more remotely.
3. Fragility fracture while on long-term GC use (≥ 3 months of prednisone-equivalent dose of 5 mg/day or above).
4. A BMD T-score ≤ -3.0 associated with any other clinical risk factor, namely, advanced age (≥ 65 years), a previous fragility fracture, high risk of falls (see above), or prolonged use of GCs.
5. Patients with fracture risk 1.2 times above the age-specific intervention threshold, as assessed by FRAX Brazil calculated with or without BMD.

To determine if a woman falls into this very−high-risk category, vertebral imaging should be obtained. Both anabolic agents available in Brazil − TPTD and romosozumab − can be used as first-line treatment in patients at very high risk of fracture, apart from those on prolonged use of GCs and fractures. In these patients, TPTD should be preferred since the use of romosozumab in GIO has not yet been established.

### Women previously treated with bisphosphonates

In patients who have been on long-term bisphosphonate use, one can consider switching to bone formation therapy if any of the following is present:

1. Women with adequate compliance and in whom secondary causes of osteoporosis have been ruled out, who continue to lose bone and sustain a fragility fracture, or those who sustain two or more fragility fractures.
2. Women with GIO who sustain a fracture while on bisphosphonate use with adequate compliance.

Vertebral imaging should be obtained in any woman on chronic use of bisphosphonates who presents symptoms or signs of vertebral fracture over the course of treatment, such as height loss or back pain (95). In those on prolonged use of GCs, vertebral imaging should be obtained every 6 months during the first year and then every 1-2 years (96). In patients switching from bisphosphonates to anabolic agents − with the exception of patients with GIO − romosozumab might be preferred, given the evidence of greater BMD improvements with this agent compared with TPTD (83). A transient decrease in hip BMD can be observed when transitioning from bisphosphonates to TPTD. This is, however, a recommendation based solely on BMD, and not on fracture data.

#### Women previously treated with denosumab

There are no data on fracture risk in patients treated with denosumab followed by TPTD or romosozumab. A small study has shown increased bone turnover and decreased bone mass in patients treated with denosumab followed by TPTD. Thus, despite the absence of fracture data, this position statement recommends against the use of TPTD following denosumab therapy.

Regarding the use of romosozumab after denosumab, small studies suggest that a short-term treatment of denosumab followed by romosozumab maintains or slightly increases bone mass. However, the safety of transitioning from a long-term denosumab therapy (>2.5 years) to romosozumab is unknown. Given the lack of data, this position statement makes no recommendation for or against the use of romosozumab following denosumab therapy.

### Safety profile of anabolic agents

Prolonged treatment of rodents with TPTD has been linked to increased risk of osteosarcoma, although this has not been observed in long-term surveillance of patients treated with the drug (97). Yet, TPTD is not recommended for patients with existing risk factors for osteosarcoma (including Paget’s disease of bone, unexplained elevations of alkaline phosphatase, prior skeletal radiation, or personal or family history of osteosarcoma) or in those with bone metastasis, multiple myeloma, or other hematologic malignancies (98). In addition, the safety and efficacy of TPTD in postmenopausal osteoporosis have only been established for a 2-year period in clinical trials, so the use of this medication should not exceed 24 months. TPTD can cause hypercalcemia and is contraindicated in patients with primary hyperparathyroidism or other hypercalcemic states. Finally, TPTD has been associated with hypercalciuria, dizziness, palpitations, nausea, and headache (67).

Treatment with romosozumab can lead to hypocalcemia, hypersensitivity reactions, and mild injection site reactions (87). More importantly, in the ARCH study, the risk of major adverse cardiac events (MACE) was greater in the romosozumab than in the alendronate group (17). This observation has led to the recommendation by Regulatory Medical Agencies across many countries, including the ANVISA in Brazil, to avoid the use of romosozumab in patients who have had a myocardial infarction or stroke within the preceding year (99,100).

## CONCLUSION

In conclusion, screening for osteoporosis, which includes clinical fracture risk assessment and BMD measurement, followed by pharmacological intervention in selected patients, reduces fracture risk. Recently, a step toward individualized treatment based on fracture risk stratification has been proposed. In postmenopausal women at very high risk of fracture, the use of a bone-forming agent as first-line therapy, followed by an antiresorptive agent, appears to reduce the fracture risk to a greater extent than antiresorptive drugs alone. This document proposes the definition of very high risk of osteoporotic fracture in postmenopausal women. It suggests bone-forming agents to be considered as first-line therapy for postmenopausal women presenting any of the following: a BMD T-score ≤ -2.5 associated with vertebral or hip osteoporotic fracture; multiple vertebral fractures or two or more nonvertebral osteoporotic fractures; a fragility fracture while on long-term GC use; a BMD T-score ≤ -3.0 associated with other clinical risk factors; or a fracture risk 1.2 times above the age-specific intervention threshold as assessed by FRAX. Additionally, anabolic agents should be considered in patients who fail bisphosphonate therapies, including those on long-term GC use. This new approach to osteoporosis treatment may reduce fracture-related morbidity and mortality.
